# Inflexibility of AMPK-mediated metabolic reprogramming in mitochondrial disease

**DOI:** 10.18632/oncotarget.20617

**Published:** 2017-09-01

**Authors:** Dar-Shong Lin, Shu-Huei Kao, Che-Sheng Ho, Yau-Huei Wei, Pi-Lien Hung, Mei-Hsin Hsu, Tsu-Yen Wu, Tuan-Jen Wang, Yuan-Ren Jian, Tsung-Han Lee, Ming-Fu Chiang

**Affiliations:** ^1^ Department of Pediatrics, Mackay Memorial Hospital, Taipei, Taiwan; ^2^ Department of Medicine, Institute of Biomedical Sciences, Mackay Medical College, New Taipei, Taiwan; ^3^ School of Medical Laboratory Science and Biotechnology, Taipei Medical University, Taipei, Taiwan; ^4^ Department of Pediatric Neurology, Kaohsiung Chang Gung Memorial Hospital, Chang Gung University College of Medicine, Kaohsiung, Taiwan; ^5^ Department of Medical Research, Mackay Memorial Hospital, Taipei, Taiwan; ^6^ Department of Laboratory Medicine, Mackay Memorial Hospital, Taipei, Taiwan; ^7^ Department of Neurosurgery, Mackay Memorial Hospital, Taipei, Taiwan; ^8^ Mackay Medicine, Nursing and Management College, Taipei, Taiwan; ^9^ Graduate Institute of Injury Prevention and Control, Taipei Medical University, Taipei, Taiwan; ^10^ Center for Mitochondrial Medicine and Free Radical Research, Changhua Christian Hospital, Changhua, Taiwan

**Keywords:** mitochondrial diseases, oxidative phosphorylation, bioenergetics, AMPK, metabolic inflexibility, Autophagy

## Abstract

Mitochondrial encephalomyopathy, lactic acidosis, and stroke-like episodes (MELAS) syndrome is most commonly caused by the A3243G mutation of mitochondrial DNA. The capacity to utilize fatty acid or glucose as a fuel source and how such dynamic switches of metabolic fuel preferences and transcriptional modulation of adaptive mechanism in response to energy deficiency in MELAS syndrome have not been fully elucidated. The fibroblasts from patients with MELAS syndrome demonstrated a remarkable deficiency of electron transport chain complexes I and IV, an impaired cellular biogenesis under glucose deprivation, and a decreased ATP synthesis. In situ analysis of the bioenergetic properties of MELAS cells demonstrated an attenuated fatty acid oxidation that concomitantly occurred with impaired mitochondrial respiration, while energy production was mostly dependent on glycolysis. Furthermore, the transcriptional modulation was mediated by the AMP-activated protein kinase (AMPK) signaling pathway, which activated its downstream modulators leading to a subsequent increase in glycolytic flux through activation of pyruvate dehydrogenase. In contrast, the activities of carnitine palmitoyltransferase for fatty acid oxidation and acetyl-CoA carboxylase-1 for fatty acid synthesis were reduced and transcriptional regulation factors for biogenesis were not altered. These results provide novel information that MELAS cells lack the adaptive mechanism to switch fuel source from glucose to fatty acid, as glycolysis rates increase in response to energy deficiency. The aberrant secondary cellular responses to disrupted metabolic homeostasis mediated by AMPK signaling pathway may contribute to the development of the clinical phenotype.

## INTRODUCTION

More than 50% of mitochondrial DNA (mtDNA) mutations resulting in diseases characterized by a broad spectrum of clinical symptoms and multi-system involvement are located in 22 tRNA genes [1]. The A to G transition at nucleotide position 3243 (A3243G) in the mitochondrial *tRNA*^*Leu(UUR)*^ gene (MT-TL1) has been reported to cause mitochondrial encephalomyopathy, lactic acidosis, stroke-like episodes (MELAS) and maternally inherited diabetes and deafness [2, 3]. The A3243G mutation is one of the most common mtDNA mutations, accounting for over 80% of all the reported cases of MELAS [2]. While the molecular impact of the A3243G mutation is still controversial, the deficiency of electron transport chain (ETC) complexes I and IV observed in MELAS cells harboring the A3243G mutation has been reported to be associated with impaired energy production [4, 5]. The insufficiency of ATP production to meet the energy demands of various organs results in multi-system disorders which may be responsible for the phenotypes observed in MELAS syndrome [6].

To accommodate the energy deficiency, reconfiguration of mtDNA and nuclear DNA (nDNA) expression profiles is induced. In the muscle biopsies of patients with MELAS syndrome, the transcript levels of mtDNA- and nDNA-encoded oxidative phosphorylation (OXPHOS) genes and of several associated bioenergetic genes have been reported to be correlated with the percentage of A3243G mutation [7, 8]. Similarly, analyses of whole blood transcriptomes from patients with MELAS syndrome revealed significant correlations between A3243G mutation load and phenotypic manifestations, as well as levels of nuclear modifier genes involved in nucleic acid and protein metabolism [9]. Furthermore, continuous changes in A3243G heteroplasmy in cybrid cells has been reported to result in abrupt alterations in both signal transduction and epigenetic regulatory processes involving profiles associated with energy metabolism, transmembrane signal transduction, senescence and telomere maintenance [10]. Taken together, the process of reconfiguration of nuclear gene expression profiles to adapt mitochondrial dysfunction plays a pivotal role in the aberrant phenotype of MELAS syndrome and will assist in the development of new therapies to treat this syndrome. Nonetheless, the capacity to utilize fatty acid or glucose as a fuel source and how such dynamic switches of metabolic fuel preferences and transcriptional modulation of adaptive mechanism in response to energy deficiency in MELAS syndrome have not been fully elucidated. In this study, we analyzed the *in situ* bioenergetic properties and ATP synthesis of the human skin fibroblasts harboring the A3243G mutation and further elucidated the transcriptional reconfiguration adapting to mitochondrial dysfunction in MELAS syndrome. Our results demonstrated a simultaneous occurrence of both impaired mitochondrial respiration and decreased fatty acid oxidation (FAO), a metabolic inflexibility in the compensation for energy deficiency and the AMP-activated protein kinase (AMPK) signaling pathway as underlying the reconfiguration of energy metabolism.

## RESULTS

### Deficiency of respiratory chain complex and decreased cell proliferation

**Figure 1:**
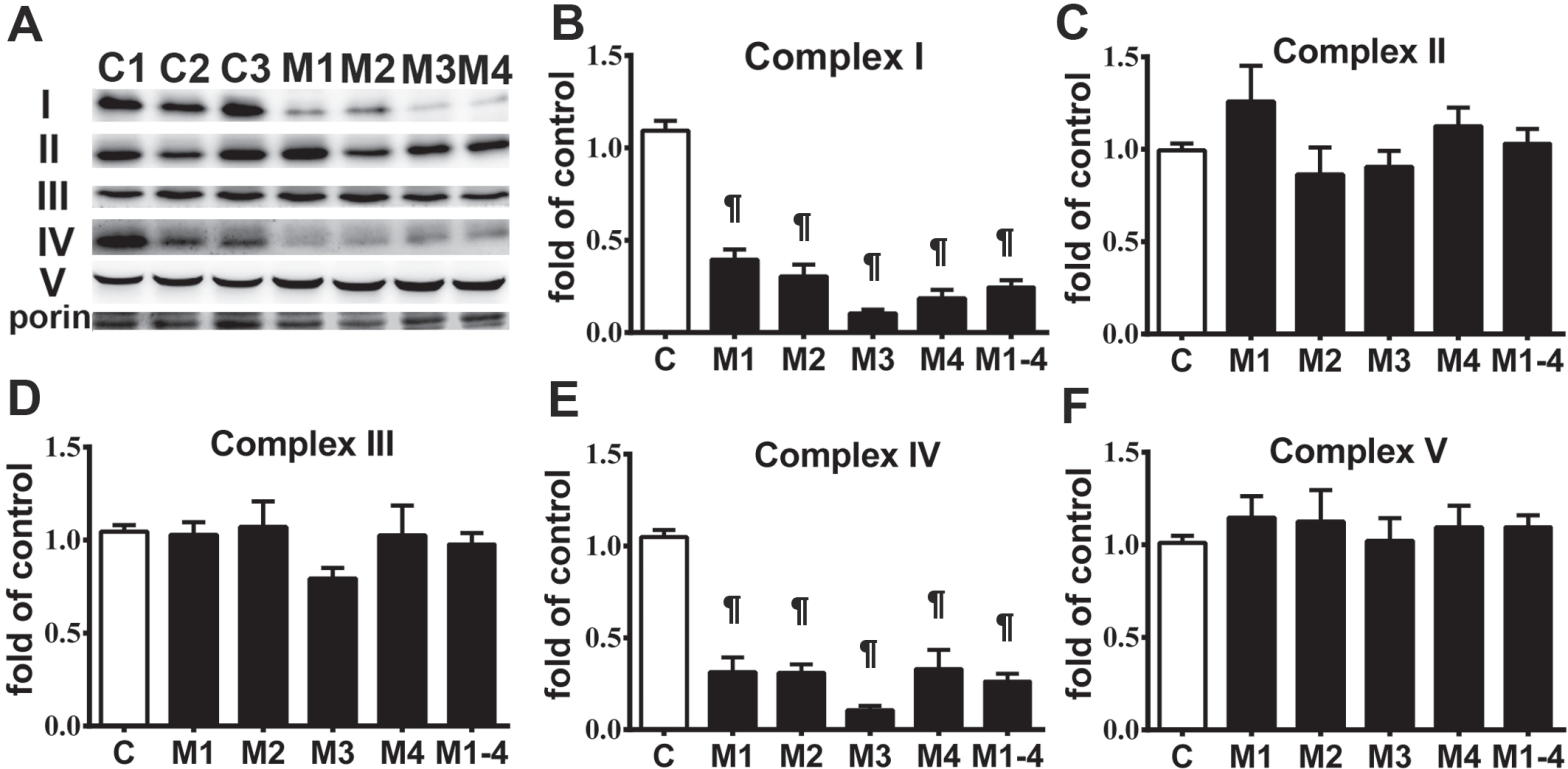
Respiratory enzyme complex deficiency in MELAS fibroblasts A representative Western blot image demonstrates the levels of respiratory enzyme complex proteins in MELAS fibroblasts (M1∼M4) and control cells **A.** Quantification results of respiratory enzyme complex proteins are presented as fold of normal **B.**-**F.** Data are expressed as mean ± SEM. *n* = 4. Three control cell lines were analyzed. C, controls; M, MELAS.* *p* < 0.05, # *p* < 0.01, ¶ *p* < 0.001.

**Figure 2:**
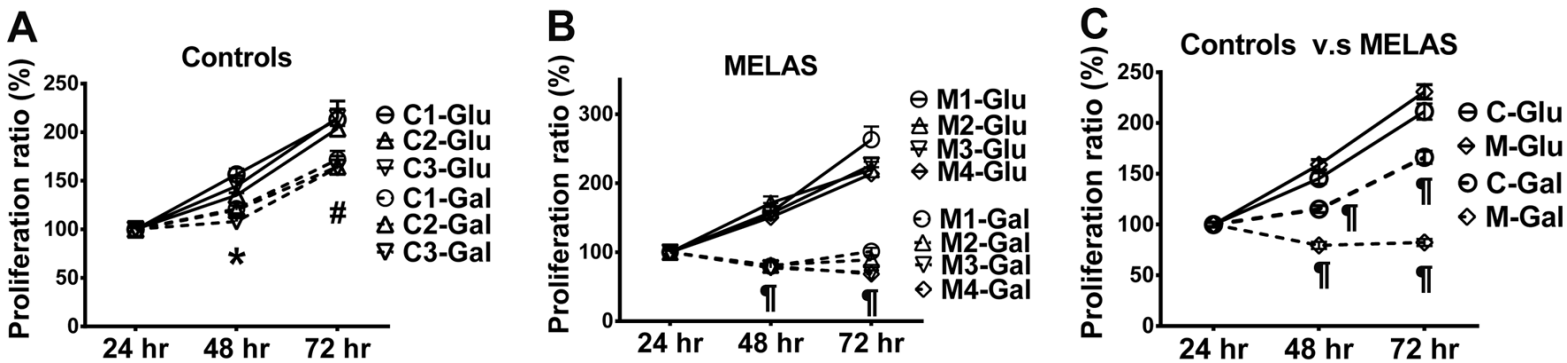
Decrease in cell proliferation of MELAS fibroblasts Cell proliferation was assayed for control **A.** and MELAS **B.** fibroblasts cultured in glucose- and galactose-containing media, respectively. Cell proliferation of MELAS fibroblasts was compared to that of controls **C.** Data are expressed as mean ± SEM. *n* = 6-9. Three control cell lines were analyzed. C, controls; Glu, glucose; Gal, galactose; M, MELAS.* *p* < 0.05, # *p* < 0.01, ¶ *p* < 0.001.

Skin fibroblasts from the patients with MELAS syndrome revealed a high level of A3243G mutation in the tRNA^Leu(UUR)^ gene (M1:93.14 %, M2: 91.70 %, M3: 95.72 % and M4: 94.77 %). Analysis of ETC complexes was performed on whole cell lysates of the fibroblasts using Western blotting (Figure 1A-1F). Quantification of ETC complexes demonstrated a marked reduction of Complex I subunit NDUFB8 (0.25 ± 0.06 of control) and Complex IV subunit MTCO2 (0.26 ± 0.04 of control) in the MELAS cells compared with the controls (Figure 1B, 1E). Subunits NDUFB8 and MTCO2 are crucial for the assembly of the membrane arm of Complex I and catalytic core of Complex IV, respectively [11, 12]. Deficiency of subunits NDUFB8 and MTCO2 resulted in reduced abundance, stability and activity of Complexes I and IV, respectively [11, 12]. Cell proliferation was assessed in the fibroblasts cultured in a glucose medium and a galactose medium, respectively. The cells derived from both the control (Figure 2A) and MELAS patients (Figure 2B) showed significantly decreased cell proliferation in the galactose medium (control cells 115.17 % ± 3.45 % and 166.22 % ± 5.90 %, MELAS cells 79.47 % ± 3.42 % and 82.30 % ± 3.37 %, at 48 and 72 hrs, respectively) compared to those cultured in the glucose medium (control cells 145.26 % ± 5.16 % and 211.26 % ± 7.84 %, MELAS cells 158.93 % ± 5.03 % and 230.77 % ± 6.98 %, at 48 and 72 hrs, respectively), while cell proliferation of the MELAS cells in the glucose medium was indistinguishable from that of the control cells (Figure 2C). Galactose forces cells to have an increased reliance on OXPHOS for energy production and is useful in studies of mitochondrial dysfunction. The MELAS cells were significantly more sensitive to the galactose medium, suggesting an impaired cellular biogenesis due to mitochondrial dysfunction.

### Bioenergetic function defects in the MELAS cells

**Figure 3:**
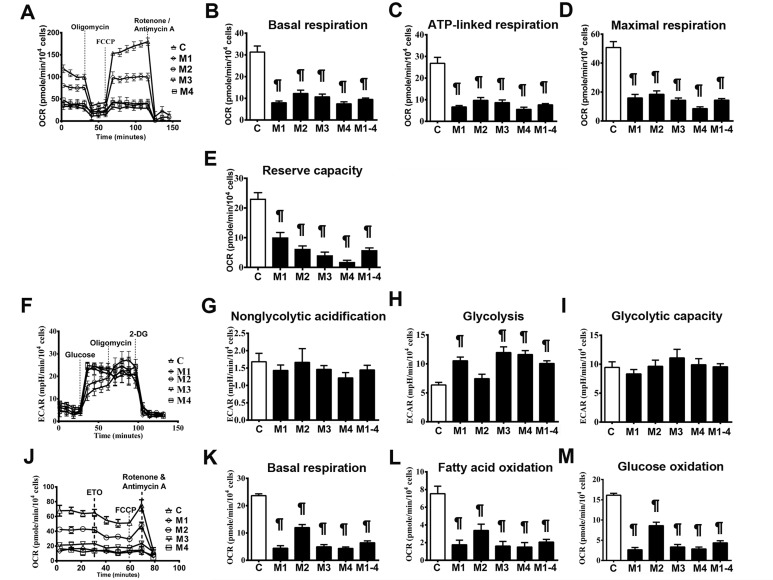
Impairment of bioenergetics in MELAS fibroblasts A representative mitochondrial function was determined by oxygen consumption rate **A.** Basal respiration rate **B.**, ATP-linked respiration rate **C.**, maximal respiration rate **D.**, and reserve capacity **E.** were measured after the sequential injections of inhibitors. A representative glycolysis function was determined by extracellular acidification rate **F.** Non-glycolytic acidification **G.**, glycolysis **H.**, and glycolytic capacity **I.** and were measured after sequential injections of glucose and inhibitors. A representative beta-oxidation of fatty acids was determined by oxygen consumption rate **J.** Basal respiration **K.** in the presence of substrate-limited medium supplemented with palmitate, and fatty acid oxidation **L.** and glucose oxidation **M.** were measured after sequential injections of inhibitors. Data are expressed as mean ± SEM. *n* = 9-15. Three control cell lines were analyzed. C, controls; 2-DG, 2-deoxy-D-glucose; ETO, Etomoxir; M, MELAS. * *p* < 0.05, # *p* < 0.01, ¶ *p* < 0.001.

We profiled the *in situ* bioenergetic properties of the MELAS cells in substrate-conditioned medium using a Seahorse XF24 extracellular flux analyzer. Oxygen consumption rate (OCR) and extracellular acidification rate (ECAR) values were recorded to measure mitochondrial respiration and glycolysis, respectively. The mitochondrial bioenergetic function (Figure 3A) in the presence of a full set of energy substrates (glucose, glutamine and pyruvate) was determined by measuring baseline and subsequent OCR after the sequential addition of the metabolic modulators oligomycin, carbonyl cyanide 4-(trifluoromethoxy) phenylhydrazone (FCCP ), and rotenone/antimycin A. Parameters of mitochondrial function including basal respiration, ATP-linked respiration, maximal respiration and reserve capacity were also determined [13]. MELAS cells showed significantly lower OCR levels of basal respiration (9.50 ± 0.61) than the controls (31.31 ± 2.79) (Figure 3B). The OCR levels of ATP-linked respiration in the MELAS cells (7.63 ± 0.56) was also significantly lower than the controls (26.79 ± 2.79) (Figure 3C). In addition, maximal respiration was significantly decreased in the MELAS cells (14.40 ± 1.10) compared to the control (50.75 ± 4.10) (Figure 3D). Furthermore, MELAS cells (5.82 ± 0.72) had ∼ 25% of the normal level (23.16 ± 2.00) of reserve capacity (Figure 3E). Overall, a decrease in mitochondrial function indicators in the MELAS cells indicated the impairment of oxidative phosphorylation, which is consistent with the deficiency in ETC complexes. Although all the MELAS cells demonstrated a decrease in bioenergetic function, the expression level among the cell lines varied, suggesting the impact of genetic background upon modulation of metabolic flexibility in response to metabolic demand.

To further characterize the glycolysis properties of the MELAS cells, glycolytic profiles (Figure 3F) were generated by measuring changes in ECAR under the sequential additions of glucose, oligomycin and 2-deoxyglucose (2-DG), respectively [14]. The non-glycolytic acidification was similar in MELAS cells (1.44 ± 0.13) and the controls (1.68 ± 0.24) (Figure 3G). The MELAS cells (10.07 ± 0.47) demonstrated an increase of glycolysis compared to the controls (6.35 ± 0.45) (Figure 3H). While the MELAS cells (9.55 ± 0.52) showed a trend of increase in glycolytic capacity compared with the controls (9.45 ± 0.97) although the difference was not significant (Figure 3I), suggesting the preferential dependence on glycolysis for the supply of ATP.

To determine the metabolic reliance on fatty acids in the MELAS cells, fatty acid oxidation profiles (Figure 3J) were generated by measuring OCR after the sequential additions of bovine serum albumin (BSA)-palmitate, etoxomir, FCCP and rotenone/antimycin A, respectively [15]. The MELAS cells (6.42 ± 0.68) showed significantly lower basal respiration compared to the controls (23.66 ± 0.69) (Figure 3K). Of note, the MELAS cells (2.06 ± 0.30) had significantly lower levels of OCR derived from fatty acid oxidation compared to the controls (7.53 ± 0.85) (Figure 3L), suggesting the underutilization of fatty acid oxidation for ATP production in the MELAS cells. Furthermore, OCR in the MELAS cells (4.36 ± 0.52) during glucose oxidation was significantly lower than that in the controls (16.13 ± 0.47) (Figure 3M), consistent with the observation of a deficiency in ETC complexes in patients with MELAS syndrome.

### Alteration of metabolic regulators

**Figure 4:**
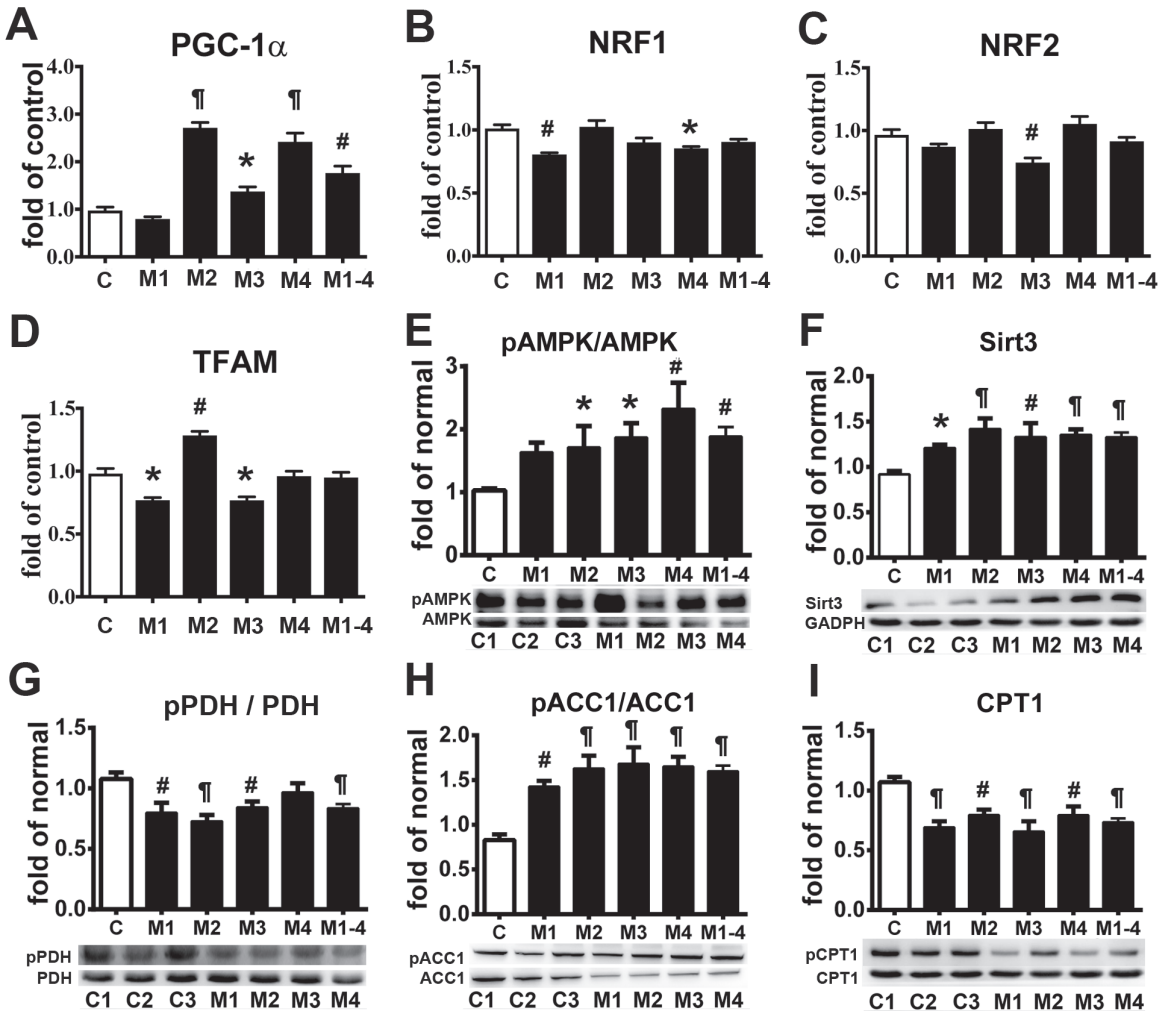
Adaptation of energy deficiency in MELAS fibroblasts is modulated through signaling pathway PGC-1α **A.**, NRF-1**B.**, NRF-2 **C.**, and TFAM **D.** mRNA levels in MELAS fibroblasts relative to controls. Western blot was performed to determine the protein expression levels of pAMPK **E.**, Sirt3 **F.**, pPDH **G.**, pACC1 **H.**, and CPT1 **I.** Data are expressed as mean ± SEM. *n* = 4 (qRT-PCR) or 6 (immunoblot). Three control cell lines were analyzed. C, controls; M, MELAS. * *p* < 0.05, # *p* < 0.01, ¶ *p* < 0.001.

Deficiency of ETC complexes I and IV in MELAS cells impairs OXPHOS function, leading to defects in energy metabolism [4, 5]. To better elucidate the cellular adaptation to impaired OXPHOS in MELAS cells, key molecules related to energy metabolism and mitochondrial function were analyzed. Quantitative RT-PCR analysis of peroxisome proliferator-activated receptor gamma coactivator 1-alpha (PGC-1α) (Figure 4A) showed a significant increase in the MELAS cells (1.76 ± 0.14 of control) compared with the control cells. Given that PGC-1α is also a key transcriptional co-activator of mitochondrial biogenesis [16], the upregulation of PGC-1α may augment this cellular process. We then quantified the mRNA expression levels of the PGC-1α downstream targets: nuclear respiratory factor 1 (NRF-1) (Figure 4B), NRF-2 (Figure 4C) and mitochondrial transcription factor (TFAM) (Figure 4D). The expression of TFAM was not significantly different between the MELAS cells (0.95 ± 0.04 of control) and the controls. Moreover, the expression of NRF-1 (0.91 ± 0.02 of control) and NRF-2 (0.91 ± 0.03 of control) were comparable between the MELAS cells and control cells. The mtDNA content was similar in the MELAS cells compared with the controls (data not shown).

To further investigate the modulation of mitochondrial bioenergetics in MELAS, we examined the protein expressions of key regulators involved in the energy metabolic pathway. There was a significant increase in Thr172-phosphorylated AMPK (pAMPK) in the MELAS cells (1.88 ± 0.15 of control) compared to the controls as determined by the pAMPK levels normalized to the total AMPK levels (Figure 4E). We further compared the protein expression of Sirtuin-3 (Sirt3) between the MELAS and control cells and found that the Sirt3 levels were significantly increased in the MELAS cells (1.33 ± 0.05 of control) (Figure 4F). These findings demonstrated that the AMPK/PGC-1α/Sirt3 signaling pathway was upregulated in the MELAS cells.

Normal fuel homeostasis in human cells involves reciprocal interplay between glucose and fatty acid metabolism. To further investigate whether the attenuated fatty acid oxidation in the MELAS cells increased glucose catabolism, we assayed the key enzymes involved in the glycolytic pathway. Pyruvate dehydrogenase (PDH) is one of the enzymes in the PDH complex which provides a link between glycolysis and the tricarboxylic acid (TCA) cycle [17]. PDH converts pyruvate to acetyl-CoA which enters the TCA cycle and generates NADH and FADH_2_ during oxidative reactions for subsequent ATP synthesis. Phosphorylation of PDH leads to its inactivation, thus inhibiting the catabolism of pyruvate to acetyl-CoA resulting in further oxidative metabolism thereby directing pyruvate to gluconeogenesis. Interestingly, we observed a significant decrease in phosphorylation of PDH in the MELAS cells (0.84 ± 0.04 of control) (Figure 4G), suggesting enhanced activity of PDH which would promote pyruvate influx into the TCA cycle [17].

The bioenergetic profile showed an attenuation of fatty acid oxidation in the MELAS cells. We then assayed the key regulators involved in fatty acid metabolism. Acetyl-CoA carboxylase 1 (ACC1) is the rate-limiting enzyme for fatty acid synthesis, and AMPK plays a pivotal role in regulating fatty acid oxidation by phosphorylating ACC1 to inactivate its lipogenic activity [18]. We measured the protein levels of ACC1 phosphorylation and observed a significantly increase in phosphorylated-ACC1suppression of ACC1 activity (1.60 ± 0.07 of control) and a decrease in total ACC1 in the MELAS cells compared to the controls (Figure 4H), suggesting the decreased fatty acid synthesis in MELAS cells was in response to a low level of cellular energy. Given that glucose oxidation is impaired in MELAS cells, it is expected that there would have been a dynamic switch of fuel preference from glucose to fatty acid. We then determined the regulatory enzymes for fatty acid oxidation. Carnitine palmitoyltransferase 1 (CPT1) is a rate-limiting enzyme that transports cytosolic long-chain acyl CoA molecules into the mitochondria for fatty acid oxidation [18]. Interestingly, the protein expression of CPT1 was significantly attenuated in the MELAS cells (0.73 ± 0.03 of control) compared to the controls (Figure 4I). This suggests that the fatty acid oxidation for energy production was not enhanced but rather attenuated in the MELAS cells under energy deficiency.

### Deficiency of ATP production

**Figure 5:**
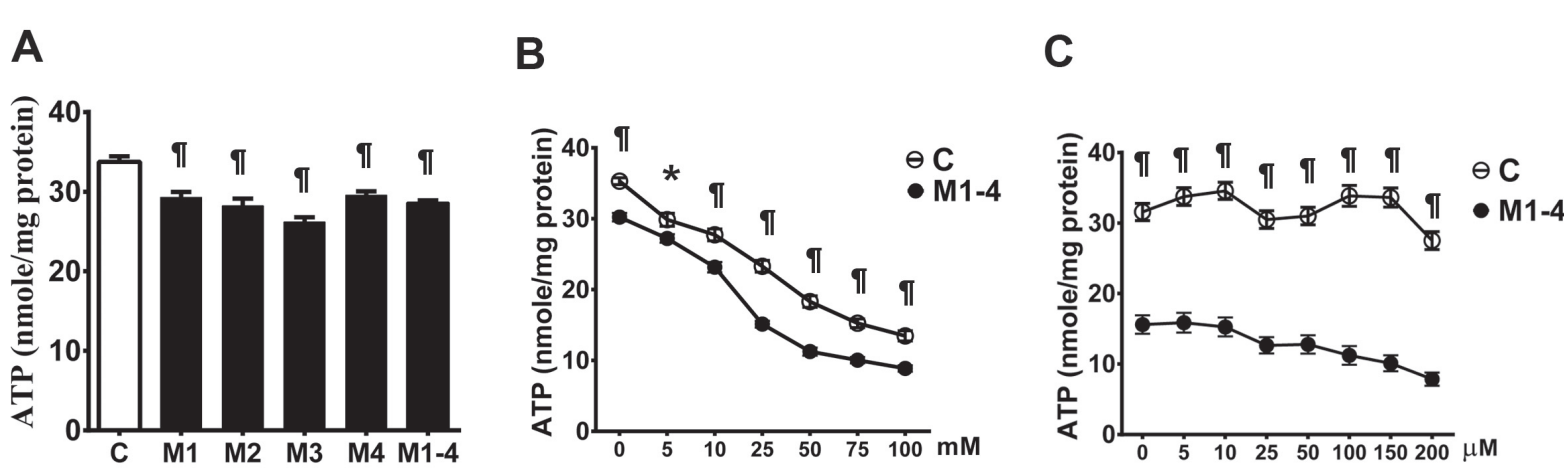
Decrease in the ATP production in MELAS fibroblasts ATP production of cells incubated in glucose medium **A.**, after the addition of glycolysis inhibitor 2-deoxy-D-glucose **B.**, and after the addition of fatty acid oxidation inhibitor Etomoxir **C.**. Data are expressed as mean ± SEM. *n* = 6-9. Three control cell lines and four MELAS cell lines (M1-M4) were analyzed. C, controls; M, MELAS. * *p* < 0.05, # *p* < 0.01, ¶ *p* < 0.001.

Cellular ATP production in the MELAS cells cultured in glucose medium with and without the addition of an inhibitor to block glycolysis or fatty acid oxidation was determined. Levels of ATP production in the MELAS cells (28.6 ± 0.37) was significantly lower than in the controls (35.75 ± 0.67) when cultured in the glucose medium (Figure 5A). We then determined ATP production with inhibition of glycolysis by 2-DG (Figure 5B) and inhibition of fatty acid oxidation by etomoxir (Figure 5C), and found that the MELAS cells were significantly sensitive to inhibition of glycolysis and fatty acid oxidation as compared to the controls.

## DISCUSSION

The A3243G mutation of mtDNA leads to a deficiency in ETC complexes I and IV, and thereby impairs the production of ATP *via* OXPHOS. The A3243G mutation prevents the taurine-modification at the wobble U base, impairing translation at UUA and UUG codons, resulting in amino acid mis-incorporation and deficiency of amino-acylation of *tRNALeu*^*(UUR)*^ [19, 20]. These changes lead to a deceased level of mitochondrial protein synthesis, severe assembly defects and enzyme activities of ETC complexes I and IV, and impaired respiratory function [4, 20, 21]. Our findings were consistent with a recent study showing significant low levels of mitochondrial function in inducible pluripotent stem (iPS) cells derived from patients with MELAS syndrome with the heteroplasmic mutation A3243G and fibroblast cells differentiated from MELAS-iPS cells [22]. Moreover, we found a switch of energy metabolism from mitochondrial respiration to glycolysis as an adaptive response to energy deficiency in MELAS cells. This is in accordance with previous studies showing a modified metabolic pathway toward a high glycolytic rate to generate ATP at the expense of increased lactate production in cybrid cells harboring A3243G mutation [23]. A recent study using myoblasts also demonstrated an increase in glucose uptake and glycolytic ATP production flux in the presence of an OXPHOS inhibitor, indicating a compensatory mechanism for a reduction in mitochondrial ATP production [24]. The dysfunction of mitochondria causes a reduction in oxidative ATP synthesis flux, alterations of metabolite homeostasis and glycolysis, which underline the molecular mechanism of mitochondrial myopathy and mitochondrial diabetes [23, 25].

To our knowledge, this is the first study demonstrating a concomitant occurrence of bioenergetic dysfunction of fatty acid oxidation and OXPHOS defects of mitochondria. To date, concurrent defects in both OXPHOS and fatty acid oxidation pathways expressed in clinical manifestations and biochemical abnormalities were observed sporadically in patients harboring either an OXPHOS defects or fatty acid metabolism disorder [26]. *In vitro* studies using cells from patients with an isolated ETC complex defect have shown impaired fatty acid oxidation and defective acylcarnitine profiles underlying the reported clinical phenotype associated with asymptomatic secondary inhibition of fatty acid oxidation [27-29]. However, the molecular mechanisms modulating concurrent OXPHOS and fatty acid oxidation defects in MELAS syndrome have not been fully elucidated. It is possible that energy deficiency caused by OXPHOS defects activates improper signaling pathways, thereby leading to metabolic perturbations which leads to failure to maintain homeostasis and this results in the clinical phenotype [26].

In the current study, AMPK activation was found to act as a mediator of an adaptive response to energy deficiency in MELAS cells through enhanced glycolysis for ATP generation. This is in accordance with previous metabolic profiling experiments identifying AMPK-mediated metabolic plasticity through enhanced glycolytic flux upon knockdown or inhibition of the OXPHOS machinery [30]. AMPK activation facilitates restoration of cellular energy status by switching on a catabolic pathway to generate ATP while simultaneously inhibiting ATP-consuming processes such as cell proliferation and biosynthesis [20].

The transcriptional coactivator PGC-1α, a downstream target of AMPK, regulates mitochondrial biogenesis by activation of the downstream targets NRF-1 and NRF-2, which in turn regulate the nuclear genes involved in the expression, assembly and function of respiratory enzyme complexes and activate TFAM to increase mtDNA replication [16]. This results in an increased expression of PGC-1α that activates the transcription of genes involved in metabolic homeostasis [31]. Interestingly, we did not observe concomitant alterations of downstream targets NRF-1, NRF-2 and TFAM with an upregulated expression of PGC-1a in MELAS cells. However, a significant increase in the expression of Sirt3 protein was observed. Sirt3, a major mitochondrial NAD^+^-dependent deacetylase, modulates mitochondrial energy catabolism and antioxidants in response to a variety of stresses [32]. The PGC-1α/Sirt3 axis is essential for the regulation of mitochondrial metabolism, bioenergetics and oxidative stress [33, 34]. Higher expressions of PGC-1α and Sirt3 proteins and increased AMPK-α phosphorylation have shown to promote OXPHOS function [35], and increase antioxidant defenses thereby modulating metabolic homeostasis and mitochondrial respiration [33, 35]. Furthermore, Sirt3 enhances mitochondrial bioenergetic function and glycolysis through the activation of PDH and lactate dehydrogenase [36, 37], while deletion of Sirt3 impairs PDH activity and augments fatty acid oxidation at the expanse of glucose oxidation [38]. It has been shown that Sirt3 regulates metabolic flexibility by optimizing an intricate switch in fuel utilization between glucose and lipid oxidation *via* reversible activation of the PDH activity [38]. Moreover, recent studies concerning energy metabolism in cardiomyocytes identified phosphorylation rather than acetylation as the mechanism modulating PDH activity [39].

In the present study, we observed an enhanced PDH activity in MELAS cells by AMPK/PGC-1α/Sirt3 pathway. Previous studies have shown that deletion of AMPK affects cardiolipin homeostasis and oxidative capacity with intact ETC content, resulting in enhanced PDH activity and glycolytic flux and decreased FAO in response to metabolic challenges [40, 41]. These studies and our results indicate that enhanced PDH activity and glycolytic flux occur when mitochondrial respiration is impaired with/without a concomitant decrease of ETC content in response to metabolic demand. An enhanced PDH leads to an increased flux of acetyl-CoA converted from pyruvate feeding into the TCA cycle for energy production or into the cytosol for *de novo* lipogenesis [17]. However, in the present study, inhibition of both fatty acid synthesis and FAO through inactivating ACC1 and a decrease of CPT1, respectively, was observed. These findings suggest a failure to adapt a metabolic reprogramming by switching fuel preference from glucose to fatty acid oxidation upon blunted glucose oxidation [18]. The attenuation of FAO in MELAS cells may be partially explained by the association with Complex I deficiency. In a murine model of Complex I deficiency, metabolomic analysis revealed that an imbalance of the NAD^+^/NADH ratio inside mitochondria results in concomitant inhibition of FAO [42]. Furthermore, it has been proposed that supercomplexes composed of Complexes I, III and IV interact with FAO enzymes and form FAO-ETC hypercomplexes to facilitate the convergence of metabolic fluxes from different pathway [43, 44]. Nonetheless, the defect in Complex I potentially disrupts FAO-ETC hypercomplexes, thus reducing the efficiency of metabolic channeling through FAO and ETC and this leads to dysfunction of both FAO and OXPHOS [44]. Accordingly, subsequent destabilization of FAO-ETC supercomplexes by Complex I deficiency contributes to the pathogenesis of combined FAO and OXPHOS clinical phenotypes [45]. Taken together, the ATP depletion caused by impairment of glucose oxidation in the ETC system does not activate fatty acid oxidation, but rather increases glycolysis for energy production in MELAS cells. This continuous surge of glycolysis in compensation for energy deficiency is consistent with the findings of a previous study in which cybrid cells harboring the A3243G mutation modified metabolic pathways with a 2.8-fold increase in glycolytic rate and 2.5-fold increase in lactate production compensating for an 83% reduction in glucose oxidation [23]. Furthermore, analysis of fluxes through the respiratory pathway in cells with inhibited OXPHOS have revealed dramatically increased flux from glycolysis and the TCA cycle for energy production [46, 47]. In addition, the increased uncoupling of glycolysis from glucose oxidation can lead to a subsequent accumulation of lactate and protons and this may underlie the mechanism of the clinical phenotype in MELAS syndrome [48].

Metabolic flexibility is the capacity of an organism to adapt fuel utilization to fuel availability [49]. In the current study, we demonstrated metabolic inflexibility in MELAS cells, in that they failed to switch substrate utilization from glucose oxidation to fatty acid oxidation in response to energy deficiency. The aberrant secondary cellular responses to disrupted metabolic homeostasis may contribute to the development of the clinical phenotype. However, restoration of a secondary cellular response may be able to alleviate the clinical phenotype [26]. This is supported by the studies using Complex I-deficient cultured skin fibroblast cells in which treatment with nicotinic acid abrogated the conflicting anabolic and catabolic networks, restored metabolic balance and improved respiratory capacity without altering the severity of OXPHOS deficiency [50]. Similar effects were observed in a murine model of Leigh syndrome by Johnson et al. [51], in which the daily treatment of the mice with rapamycin rescued the metabolomic defects associated with Complex I deficiency, alleviated neurological symptoms and extended the lifespan of the mice. Further studies in the mice harboring mtDNA deletions showed that a ketogenic diet normalized the aberrant catabolism and anabolism without affecting mtDNA deletion load [51]. These findings suggest that secondary cellular signaling is a potential therapeutic target to correct aberrant metabolism and prevent the clinical phenotype for development of effective treatment of mitochondrial diseases.

**Figure 6:**
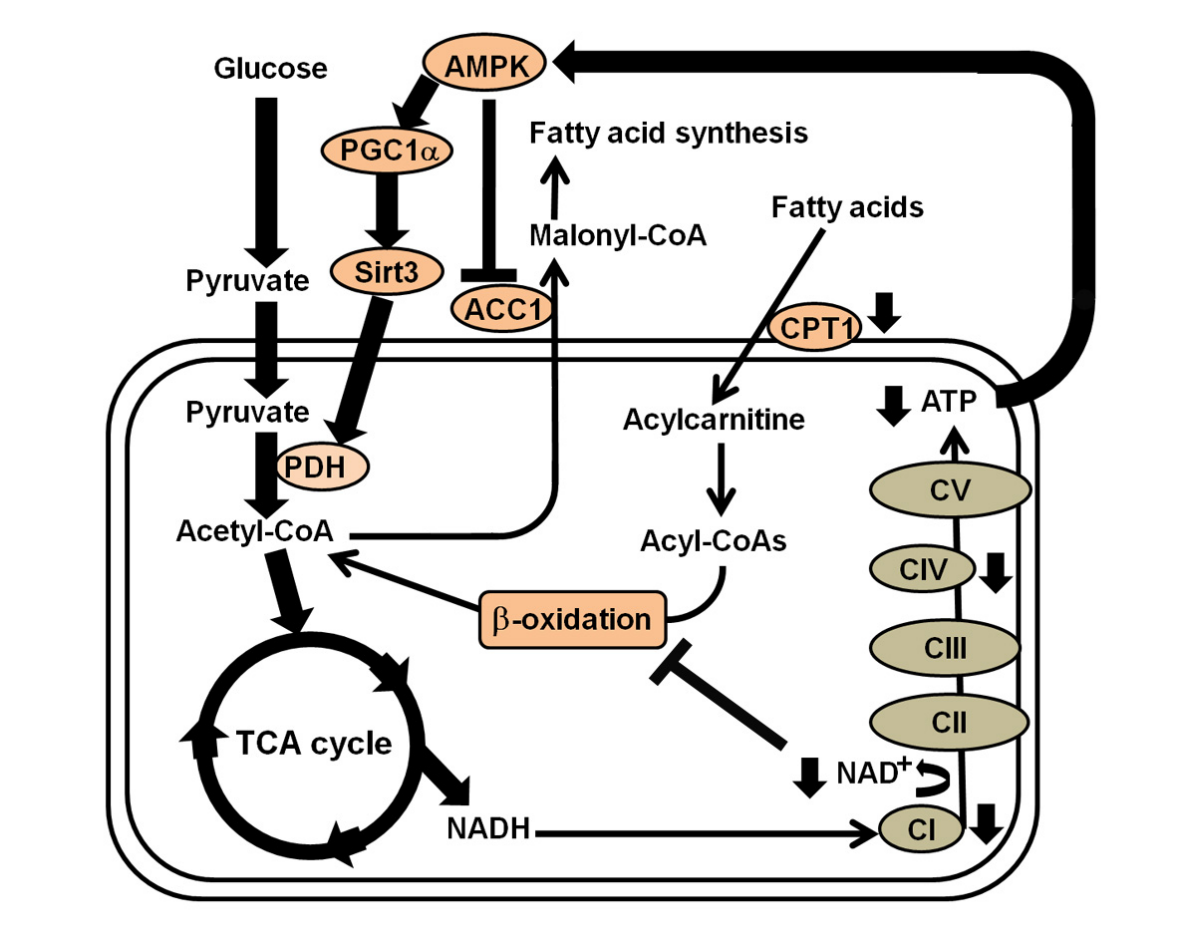
Summary of the transcriptional modulation of metabolic inflexibility in the fibroblasts of patients with MELAS syndrome Energy depletion caused by electro transport chain complexes I and IV defects in MELAS syndrome activates the AMPK/PGC-1α/Sirt3 signaling pathway, which turns on the catabolic pathway and switches off anabolic metabolism, whereas Complex I deficiency and NAD^+^/NADH imbalance reduces efficiency of fatty acid oxidation. Thus, an increase in PDH leads to an increased flux of acetyl-CoA into the TCA cycle, while a decrease in ACC1 and CPT1 leads to inhibition of fatty acid synthesis and reduction of fatty acid oxidation, respectively.

Taken together, this is first study describing the *in situ* bioenergetic profiles that connect mitochondrial function, glycolysis and fatty acid oxidation and the secondary signaling mechanism that underly the metabolic inflexibility in MELAS cells (Figure 6). These novel findings indicate that the impaired OXPHOS function resulting from the A3243G mutation of mtDNA does not activate an adaptive switch of energy metabolism to FAO, but rather results in a reduction in FAO and a reliance on glycolysis as an alternative way to restore energy deficiency. This cellular adaptation is modulated through a signaling network of the AMPK/PGC-1α pathway. An understanding of the links between transcriptional modulation, metabolic inflexibility and clinical phenotype may provide a proof of principle for the development of mitochondria-targeted interventions for mitochondrial diseases.

## MATERIALS AND METHODS

### Cell cultures

In compliance with the Declaration of Helsinki of the World Medical Association were approved by the Institutional Review Board of Mackay Memorial Hospital. Informed consent was obtained from each of the patients before any investigation of this study was performed. Primary cultures of skin fibroblasts were derived from patients with MELAS syndrome (M1, M2, M3, M4) in whom their mtDNA had the A3243G mutation in the MT-TL1 gene, and three healthy volunteers were recruited as normal controls. Cells were routinely grown in standard Dulbecco’s modified Eagle medium (DMEM; Invitrogen) with high glucose (4.5 g/l) supplemented with 10% (v/v) fetal bovine serum (FBS; Gibco) and 1% penicillin G/streptomycin sulfate, in a humidified atmosphere of 5% (v/v) CO_2_ at 37°C. When indicated, cells were cultured in serum-free medium for 6 hr, then in 5% (v/v) FBS medium for 24 hr. All the fibroblast cell lines were grown between the 5th and 10th passages for all experiments.

### Cell proliferation assay

Cells were plated at a density of 20,000 cells/well in 24-well tissue culture dishes for 24 hr. The culture medium was changed to DMEM medium supplemented with 25mM glucose or 10mM galactose. After 24, 48 and 72 hr, the cells were trypsinized and resuspended in medium, and then counted using a CyQUANT^®^ Cell Proliferation Assay Kit (Molecular Probe, Invitrogen). Cell proliferation ratios at 48 and 72 hr relative to that at 24 hr were determined.

### Cellular bioenergetics

Cells were plated at a density of 27,000 cells per well of an XF24 cell culture microplate (Seahorse Bioscience) in 250 µl of DMEM and incubated overnight (14-16 hr) in a humidified atmosphere of 5% (v/v) CO_2_ at 37°C. The culture medium was changed 1 hr prior to the assay run to bicarbonate-free XF assay medium supplemented with 25mM glucose, 2mM glutamate, 1mM sodium pyruvate,1x nonessential amino acid and 1% FBS, and the cells were equilibrated in a non-CO_2_ incubator. Mitochondrial OCR and ECAR were monitored in real time during each measurement cycle, which consisted of mix/wait/measure times of 30 sec/3 min/3 min on a Seahorse XF24 Analyzer (Seahorse Biosciences). The OCR and ECAR values were measured at baseline and after the sequential administration of oligomycin (1µM), FCCP (1 µM) and rotenone (0.6 μM) /antimycin A (0.36 µM). Indices of mitochondrial function including basal respiration rate, ATP-linked respiration rate, maximal respiration rate, and reserve capacity were calculated [13]. Basal respiration, calculated as the difference between baseline OCR and non-mitochondrial OCR remaining after rotenone/antimycin A inhibition, indicated the cellular oxygen consumption during ATP production in base medium supplemented with all energy substrates. ATP-linked respiration rate was determined after inhibition of mitochondrial ATP synthase by oligomycin. The subsequent addition of FCCP, which collapses the proton gradient across the inner mitochondria membrane, induced flux of uncoupled mitochondrial respiration leading to an immediate increase in OCR. Thus, the difference between OCR values obtained after FCCP stimulation and OCR values after rotenone/antimycin A inhibition was defined as maximal respiration. The difference between maximum respiration rate and basal respirations rate provides an estimate of the reserve capacity of the cells. To determine glycolytic profiles, adherent cells were equilibrated in a glucose-free assay medium without serum and bicarbonate, but supplemented with 2 mM L-glutamine for 1 hr [14]. ECAR of the glycolytic profiles was measured at baseline and after consecutive injections of glucose (10 mM), oligomycin (1 µM), and 2-DG (0.1 M). We determined glycolytic profiles including non-glycolytic acidification rate, glycolysis, and glycolytic capacity accordingly [14]. The cells were incubated in base medium lacking glucose and fetal bovine serum for 1 hr prior to the start of the experiment. ECAR measured during incubation with glucose-free medium was defined as non-glycolytic acidification. Subsequent supplements of glucose to the glucose-deprived cells triggered a glycolytic flux resulting in a robust increase in ECAR, defined as glycolysis. The subsequent addition of oligomycin inhibited mitochondrial ATP production resulting in a compensatory elevation in ECAR. The difference in ECAR values between the glucose-deprived and oligomycin-inhibited conditions was defined as glycolytic capacity. Finally, overall glycolysis was abolished after the addition of 2-DG to the assay medium. To determine the OCR due to beta-oxidation of exogenous fatty acids, adherent cells were equilibrated for 1 hr in substrate-limited medium (without serum, bicarbonate, glutamine and pyruvate) supplemented with 5.5 mM glucose and 0.5 mM carnitine. OCR of fatty acid oxidation was measured after consecutive injections of exogenous BSA-palmitate (200 µM) for 30 min followed by etomoxir (40 µM), FCCP (1 µM) and rotenone (0.6 μM) /antimycin A (0.36 µM) [15]. The capacity of FAO and glucose oxidation was assessed accordingly [15]. The basal respiration was measured after the addition of BSA-palmitate. The subsequent addition of etoxomir inhibited fatty acid oxidation resulting in a decrease in OCR, which was defined as fatty acid oxidation [17]. Following the addition of FCCP, maximum OCR was obtained. Finally, the addition of rotenone/antimycin A abolished overall mitochondrial respiration. The difference between OCR values after the addition of etoxomir and OCR values after the addition of rotenone/antimycin A was defined as that from glucose oxidation [17]. Data were normalized by cell number and expressed in pmol/min/10^4^ cells for OCR values and mpH/min/10^4^ cells for ECAR values to allow for comparisons between independent experiments.

### Western blotting

An aliquot of 50 μg of whole cell extracts was dissolved in sodium dodecyl sulfate-polyacrylamide gel electrophoresis sample buffer, and resolved on NuPAGE 4%-12% Bis-Tris Novex gels with MES buffer. After electrophoresis, proteins were transferred to a piece of polyvinylidene membrane (PVDF) in Invitrogen transfer buffer at 4 °C. The PVDF membrane was blocked with 5% non-fat milk for 1hr and incubated with 1:1000 primary antibodies for 2 hr at room temperature. The PVDF membrane was then washed, probed with horseradish peroxidase-conjugated secondary antibodies for 1 hr at room temperature, washed again, and then visualized by enhanced chemiluminescence (GE, Healthcare). The following antibodies were used: Complex I subunit NDUFB8, Complex II subunit SDHB, Complex III subunit UQCRC2, Complex IV subunit MTCO2, Complex V subunit ATP5A, porin (1:1000; Abcam); AMPKα, phospho-AMPKα (Thr172), ACC, phospho-ACC (Ser79), Sirt3, CPT-1, and PDH (1:1000; Cell Signaling); and phospho-PDHE1α (Ser293) (1:1000; Millipore).

### Real-time quantitative PCR

Total RNA was isolated from cells using a Total RNA mini kit (NoveiGene) according to the manufacturer’s protocol. cDNA was synthesized using a Revert Aid First Strand cDNA Synthesis Kit (ThermoScientific) according to the manufacturer’s instructions. Quantitative RT-PCR was performed in triplicate for each reaction using Power SYBR Green PCR Master Mix (Applied Biosystems) and an ABI- 7500 Fast Real-Time PCR System (Applied Biosystems). The primers for measuring PGC-1α, and TFAM were designed using OriGene’s proprietary primer design algorithm, while primer pairs for measuring NRF-1 and NRF-2 were designed using Primer3 software. Primer sequences are available upon request. The mRNA expression level of a desired gene was determined and normalized to that of GAPDH according to the delta-delta-Ct method.

### Measurement of intracellular ATP content

Intracellular ATP content was measured using an ATPlite™ Luminescence Assay system (PerkinElmer) according to the manufacturer’s instructions. Briefly, cells were plated at a density of 5x10^4^ cells/well in a 96-well culture plate and incubated in a humidified atmosphere of 5% (v/v) CO_2_ at 37 °C for 24 hr. The cells were exposed to glucose (25 mM), etomoxir (500 µM) and 2-DG (100 mM), respectively, in a carbonate-free medium for 45 min before assay. The plate was washed with PBS, added with 50 μl/well mammalian cell lysis solution, shaken for 5 min in an orbital shaker at 700 rpm, added with 50 μl/well substrate solution, and then shaken for 5 min in an orbital shaker at700 rpm to release the intracellular ATP. The 96-well culture plate was dark adapted for 10 min. The luminescence intensity from each well was measured using an Infinite 200 PRO micorplate reader (TECAN). The intracellular ATP content was normalized by the cell number.

### Statistical analysis

Data are presented as means ± SEM. Statistical significance across groups was determined by one-way ANOVA and/or Student’s *t*-test. A *P* value < 0.05 is considered statistically significant difference.
